# Design, characterization and in vitro evaluation of HPMC K100 M CR loaded Fexofenadine HCl microspheres

**DOI:** 10.1186/s40064-016-2322-2

**Published:** 2016-05-25

**Authors:** Paroma Arefin, Ikramul Hasan, Md. Selim Reza

**Affiliations:** Department Pharmaceutical Technology, Faculty of Pharmacy, University of Dhaka, Dhaka, 1000 Bangladesh

**Keywords:** Emulsification-solvent evaporation, Fexofenadine HCl, Microsphere

## Abstract

The aim of the current study was to formulate Fexofenadine hydrochloride loaded sustained release microspheres using HPMC K100 M CR, a release retardant hydrophilic polymer by solvent evaporation method. The effect of different drug loading on drug content, drug encapsulation efficiency and release of drug was monitored. The studies on in vitro release mechanism were performed using USP paddle method with 900 ml of phosphate buffer (pH 6.8) for 10 h at 100 rpm. The mechanism of the drug release was determined by fitting in vitro release data to various release kinetic models such as the zero order, first order, Higuchi, Hixson Crowell and Korsemeyer–Peppas model and finding R^2^ values for the release profile corresponding to each model. The results confirm that the release rate of the drug from the microspheres is highly affected by the drug to polymer ratio. The study finds that Higuchi release kinetics, Korsmeyer–Peppas release kinetics and Hixson–Crowell release kinetics were the major release mechanism. The release mechanism was found to be non-Fickian with increase of polymer content. Scanning electron microscopic technique was performed to obtain the morphological changes due to different drug loading. Differential scanning calorimetry and Fourier transform infra-red spectroscopy was performed to determine any interaction of drug with the polymer. A statistically significant variation in release rate was observed for variation in the amount of HPMC K100 M CR. In the present study, a series of sustained release formulations of Fexofenadine hydrochloride were developed with different drug loading so that these formulations could further be evaluated from the in vivo studies. The formulations were found to be stable and reproducible.

## Background

Microspheres, a potential drug delivery system in the segment of novel drug delivery, are defined as structure made up of continuous phase of one or more miscible polymers in which drug particles are dispersed at the molecular or macroscopic level. Generally the size of the microencapsulated products (micro-particles) is considered as larger than 1–1000 μm in diameter (Parida et al. 2008). Among the several different methods of microencapsulation the most common are solvent evaporation, coacervation, coalescence and phase separation, interfacial polymerization, spray drying and ionotropic gelation. Solvent-evaporation method engages emulsification of a solution of polymer and drug with an additional medium in which the drug and polymer cannot dissolve. The technique is comparatively uncomplicated and has been used for the formulation of microcapsules of a range of compounds using several polymers of different purpose (Bolourtchian et al. 2005).

Fexofenadine HCl (FFN) is a second-generation nonsedating histamine H_1_ receptor antagonist extensively used in seasonal allergic rhinitis. The usual dose for adults is 60 mg two times a day or 180 mg once a day. The half life of the drug is 14.4 h and the oral bioavailability is 30–41 % (Product Information: Allegra reviewed 1/2000). The adverse effect was reported to be anaphylaxis and hypersensitivity reactions (Product Information: Allegra reviewed 10/2001). As it is under the class III of the biopharmaceutical classification system (BCS), polymers can be used to prolong its duration of action in body, thereby increasing the bioavailability. Thus to increase the bioavailability by sustaining the action and to reduce the frequency of administration, to lessen adverse effects and to improve patient compliance, a controlled release formulation of the drug is required. Some extended release matrix tablets of Fexofenadine HCl are available in the market, but microspheres offer more consistent drug distribution and reduced dose dumping compared to matrix tablets.

This research aimed to develop polymeric microspheres of Fexofenadine HCl using HPMC K100 M CR polymer to offer sustained release delivery of the drug to aid in long term therapy with high margin of safety. HPMC K100 M CR is a hydrophilic release retardant polymerand is extensively studied for encapsulating material to develop controlled release dosage forms. Several researchers have investigated the utilization of HPMC K 100 M CR as a polymer to microencapsulate a drug utilizing emulsion solvent evaporation technique (Uddin et al. 2013; Raut et al. 2013).

## Methods

### Materials

Fexofenadine HCl was received as a generous gift from Incepta Pharmaceuticals Ltd. HPMC K100 M CR (Evonik Industries, Germany), ethanol (MERCK, Germany), *n*-hexane (MERCK, Germany), Span 80 (sorbitanmonooleate) (MERCK, Germany), light liquid paraffin (MERCK, Germany), potassium dihydrogen phosphate (MERCK, Germany) and dipotassium hydrogen phosphate (MERCK, Germany) were used in this study from the indicated sources.

### Preparation of microspheres of Fexofenadine HCl with HPMC K100 M CR polymer by solvent evaporation technique

The microspheres of Fexofenadine HCl were prepared based on the “emulsion-solvent evaporation technique” using HPMC K100 M CR. Ethanol was used as solvent. Span80 was used as lipophilic surfactant for Fexofenadine HCl water in oil (W/O) type of emulsions (Ayon et al. 2014; Raval et al. 2011; Jalil and Nixon 1989).

### Formulation design

Total five batches (each of 900 mg) of microspheres, designated as F1–F5 were prepared with Fexofenadine HCl (Table 1) using HPMC K100 M CR.

**Table 1:** Formulation protocol for the microspheres prepared by using HPMC K100 M CR with different drug loading

Formulation	Drug:polymer	Drug (mg)	Methocel K 100 M CR (mg)
F1	2:1	600	300
F2	1.5:1	540	360
F3	1:1	450	450
F4	1:1.5	360	540
F5	1:2	300	600

### In vitro characterization of Fexofenadine HCl microspheres

#### Production yield

The yield (%) was determined by the following equation (El-Kamel et al. 2006):

$${\text{Yield}}\,(\% )= \frac{{{\text{Weight}}\,{\text{of}}\,{\text{microspheres}}\,{\text{in}}\,{\text{grams}}}}{{{\text{Weight}}\,{\text{of}}\,{\text{total}}\,{\text{amount}}\,{\text{of}}\,{\text{materials}}\,{\text{added}}}} \times 100$$

#### Determination of drug entrapment efficiency

Ten milligram of accurately weighed microspheres was crushed in a glass mortar-pestle and was then suspended in 10 ml of pH 6.8 phosphate buffer solution. After 24 h the solution was filtered and the filtrate was assessed for drug content. The drug entrapment was calculated using the formula (Patel and Patel 2007):

$${\text{Entrapment}}\,{\text{efficiency}}\, (\% )= \frac{{{\text{Calculated}}\,{\text{drug}}\,{\text{concentration}}}}{{{\text{Theoretical}}\,{\text{drug}}\,{\text{concentration}}}} \times 100$$

#### Micromeritics study

Carr’s compressibility index and Hausner ratio were determined. Bulk density and tapped density were measured by a volumetric cylinder. Particles having excellent flow properties will have value of Carr’s compressibility index, Hausner ratio and angle of repose in the range of 5–10, 1.00–1.11 and 25–30 respectively (Lumay et al. 2012).

#### Surface morphology study with the help of scanning electron microscope (SEM) analysis

The shape and surface morphology of the microspheres was visualized by scanning electron microscopy (s-3400 N, Hitachi). The samples were prepared by lightly sprinkling the microspheres on a double-sided adhesive tape fixed on aluminum stubs. The stubs were then put into an ion sputter coater and coated with platinum in the inert environment of argon. After coating, the samples were arbitrarily examined by an electron beam to get 3D images of the microspheres. The effects of polymer concentration on the microsphere shape, integrity and their drug release pattern were studied.

#### In vitro dissolution study of Fexofenadine HCl microspheres

The in vitro drug release profile from various formulations of microspheres were performed in a paddle type (Type II) dissolutiona pparatus in simulated intestinal fluid (phosphate buffer solution, PBS, pH 6.8) at 100 rpm and temperature was maintained fixed at 37 °C. The dissolution process was carried out for 10 h and 10 ml dissolution sample from each dissolution media was withdrawn at a predetermined intervals of 30 min, 1st, 2nd, 3rd, 4th, 5th, 6th, 7th, 8th, 9th and 10th h and the release was measured by UV spectrophotometric method at 259 nm (Manekar et al. 1992).

### Data analysis

To analyze the in vitro release data various kinetic models were used to describe the release kinetics. The zero order rate equation (1) describes the systems where the drug release rate is independent of its concentration. The first order equation (2) describes the release from system where release rate is concentration dependent. Higuchi (1963) described the release of drugs from microspheres as a square root of time dependent process based on Fickian diffusion equation (3). The Hixson–Crowell cube root law equation (4) describes the release from systems where there is a change in surface area and diameter of particles or tablets.

1 $${\text{C}} = {\text{k}}_{ 0} {\text{t}}$$

where K_0_ is zero-order rate constant expressed in units of concentration/time and t is the time.

2 $${\text{Log}}\,{\text{C}}_{0} - {\text{Log}}\,{\text{C}} = {\text{kt/}}2.303$$

where C_0_ is the initial concentration of drug and K is first order constant.

3 $${\text{Q}} = {\text{Kt}}_{1/2}$$

where K is the constant reflecting the design variables of the system.

4 $${\text{Q}}_{0}^{1/3} {-}{\text{Q}}_{\text{t}}^{1/3} = {\text{K}}_{\text{HC}} {\text{t}}$$

where Q_t_ is the amount of drug released in time t, Q_0_ is the initial amount of the drug in tablet and K_HC_ is the rate constant for Hixson–Crowell rate equation.

### Successive fractional dissolution time

To characterize the drug release rate in different experimental conditions, time to release 25 % drug (T_25%_), time to release 50 % drug (T_50%_), time to release 50 % drug (T_80%_) and mean dissolution time (MDT) were calculated from dissolution data.

### Statistical analysis

Statistical analysis of the results was performed by using one-way analysis of variance (ANOVA) followed by Dennett’s t test for comparisons. The limit of significance was set at P < 0.05.

### Compatibility studies of drug and polymer within Fexofenadine HCl microspheres

#### Fourier transform infrared spectrophotometry (FTIR)

The IR spectrum of the pure drug, pure polymer, physical mixture of drug and polymer and optimized microsphere formulations were obtained to evaluate the chemical integrity and compatibility of the drug with the polymers in the microspheres.

#### Differential scanning calorimetery (DSC)

DSC study was carried out to evaluate the interaction between the drug and the polymers in the microspheres by using a differential scanning calorimeter (DSC 60, Shimadzu).

## Results and discussion

### Production yield (%) of microspheres

**Fig. 1:**
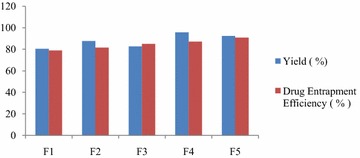
Production yield (%) and drug entrapment efficiency of microspheres

Figure 1 shows that yield did not follow any particular pattern of change with drug loading.

The practical production yield was found to be the highest for formulation F4 containing high viscosity HPMC K100 M CR (40 % drug loading). For microspheres prepared with HPMC K100 M CR, the lowest value was found to be 80.45 % for F1 (66.66 % drug loading).

### Drug entrapment efficiency (%) of microspheres

The entrapment efficiency was determined spectrophotometrically by measuring the absorbance at 259 nm using pH 6.8 phosphate buffer solution. As Fig. 1 suggests, entrapment efficiency was found to be minimum for formulation F1 (78.86 %) and maximum for formulation F5 90.94 %. It was improved with an increase in polymer concentration because a higher amount of polymer was available for cross linking which prevented drug diffusion from the microspheres (Panchaxari et al. 2013).

### Micromeritics study of microspheres prepared with HPMC K100 M CR

The microspheres exhibited good to excellent flow properties (Table 2). The true density and bulk density values were in the range of 0.31–0.47 and 0.27–0.43 g/cm^3^ respectively. The most acceptable flow ability was showed by F3 (50 % drug loading) and the worst flow properties were exhibited by F1 (66.66 % drug loading). Using these values Carr’s index and Hausner ratio were found to be in the range of 8.51–12.90 and 1.09–1.15 respectively. The result inferred that the prepared microspheres possess good to excellent flow properties.

**Table 2:** Results of micromeritics study of microspheres

Formulation	Bulk density (g/ml)	Tapped density (g/ml)	Carr’s compressibility index	Hausner ratio
F1	0.27	0.31	12.90	1.15
F2	0.39	0.43	9.30	1.10
F3	0.44	0.46	4.35	1.05
F4	0.34	0.37	8.11	1.09
F5	0.43	0.47	8.51	1.09

### Observation of particle morphology by scanning electron microscope (SEM)

**Fig. 2:**
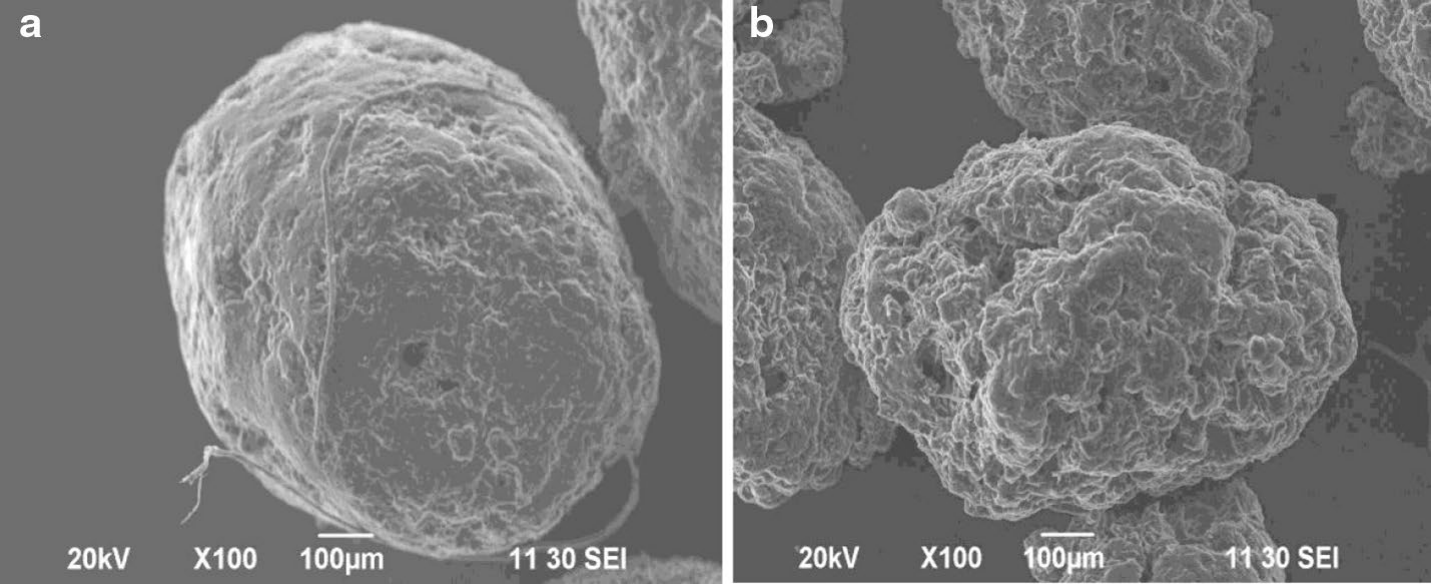
Scanning electron microscopic view of microspheres. **a** Formulation F1 (66.67 % drug loading) and **b** formulation F5 (33.33 % drug loading)

Figure 2 shows that,  maximum microspheres were found to be almost spherically shaped when observed with an optical microscope, but when observed by SEM they were found to be slightly irregular shape and rough surface which is responsible for the sustained, but earlier drug release. The surface morphology of the microspheres (F1–F4) was dependent on the concentration of polymers. Microspheres having a higher proportion of polymers were found to have a smooth surface due to the availability of more polymers for cross-linking causing the entrapment of more drug. Batch F1 (66.67 % drug loading), represented by Fig. 2a, is the microsphere with a rough and fractured surface. The shape of the microspheres of Batch F5 (33.33 % drug loading), represented by Fig. 2b, is almost spherical and the surface of the particles is smoother. There was no significant level of fusion among particles. Presence of pores or cracks may help releasing the drug since these facilitate the penetration of dissolution medium into the microsphere. Roughness of the surface increases the chances of wetting and contact of water with the microsphere than the smoother one nature of the surface influences the constancy, quality, integrity and dissolution characteristics of the microspheres. At high concentration, some of the microspheres were fused to each other, which may be due to the presence of higher amount of water, which slowly evaporates upon stirring, causing the particles to come in contact with each other.

### In vitro dissolution and kinetic studies of Fexofenadine HCl loaded polymeric microsphere

**Fig. 3:**
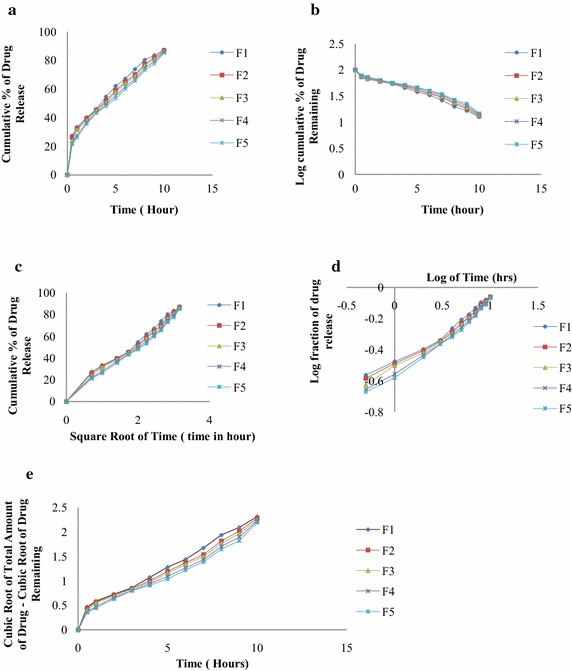
In vitro release kinetics of five formulations (F1–F5) of Fexofenadine HCl microspheres prepared with HPMC K100 M CR. **a** Zero order release, **b** first order release, **c** Higuchi plot, **d** Korsmeyer–Peppas plot, **e** Hixson–Crowell plot

HPMC K 100 M CR polymer was used as the release retardant polymer to prepare Fexofenadine HCl loaded microspheres having drug loading 33.33, 40, 50, 60, 66.66 % with the formulation F1, F2, F3, F4 and F5 respectively. After preparation according to formulation shown in the (Table 1), their dissolution studies were carried out in paddle method (USP apparatus II) in simulated intestinal fluid (pH 6.8) at 100 rpm at 37 °C. The release profile of the formulations was monitored up to 10 h. The rate of drug release was found to be inversely related to the amount of HPMC K 100 M CR present in the formulations, i.e. the rate of drug release increased with decrease in the polymer content. Figure 3 shows in vitro release kinetics of five formulations (F1–F5) of Fexofenadine HCl microspheres prepared with HPMC K100 M CR: A representing zero order release pattern, B showing first order release profile, C giving the Higuchi impact, D characterizing Korsmeyer–Peppas plot and E signifying Hixson–Crowell plot. Among all the batches, F1–F5 (Table 1), F5 showed best release retardant property. From Table 3, it can be inferred that the percent release of drug after 10 h was 85.31 % from F5, showing the highest release retarding property. Best fitted model for this formulation were Higuchi (R^2^ = 0.990), Korsmeyer–Peppas (R^2^ = 0.985) and Hixson–Crowell (R^2^ = 0.978). F1 showed least release retardant property. After dissolution of microsphere the percent release of drug after 10 h was 85.31 % from F1. First order (R^2^ = 0.983), Higuchi (R^2^ = 0.991), Korsmeyer–Peppas (R^2^ = 0.975) and Hixson–Crowell (R^2^ = 0.981) models fit best to F1. F1–F3 followed Fickian transport and F4–F5 followed non-Fickian transport (Table 4).

**Table 3:** Release rate constants and R^2^ values for different release kinetics of five formulations (F1, F2, F3, F4 and F5) of Fexofenadine HCl microspheres

Formulation	Zero order		First order		Higuchi		Korsmeyer–Peppas			Hixson–Crowell	
	K_0_	R^2^	K_1_	R^2^	K_H_	R^2^	n	K_KP_	R^2^	K_HC_	R^2^
F1	7.378	0.920	−0.187	0.983	26.48	0.991	0.403	0.33	0.975	0.206	0.981
F2	7.236	0.927	−0.177	0.974	25.86	0.989	0.406	0.32	0.976	0.197	0.979
F3	7.247	0.934	−0.173	0.973	25.84	0.992	0.428	0.30	0.983	0.194	0.980
F4	7.272	0.946	−0.168	0.968	25.77	0.991	0.453	0.28	0.986	0.191	0.980
F5	7.204	0.948	−0.161	0.964	25.46	0.990	0.462	0.27	0.985	0.187	0.978

**Table 4:** The best fitted model and mechanism of drug release from F1, F2, F3, F4 and F5

Formulation	Best fitted model	n value	Release mechanism
F1	First order, Higuchi, Korsmeyer–Peppas and Hixson–Crowell	0.403	Fickian transport
F2	First order, Higuchi, Korsmeyer–Peppas and Hixson–Crowell	0.406	Fickian transport
F3	First order, Higuchi, Korsmeyer–Peppas and Hixson–Crowell	0.428	Fickian transport
F4	Higuchi, Korsmeyer–Peppas and Hixson–Crowell	0.453	Non-Fickian/anomalous transport
F5	Higuchi, Korsmeyer–Peppas and Hixson–Crowell	0.462	Non-Fickian/anomalous transport

### Successive fractional dissolution time

**Fig. 4:**
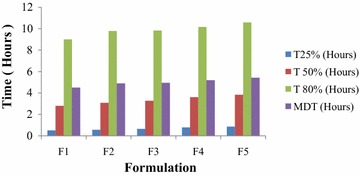
Bar diagram representing successive fractional dissolution time of five formulations *F1*, *F2*, *F3*, *F4* and *F5*

Successive fractional dissolution time was observed to be highest for F5 and lowest for F1 (Fig. 4).

### Analysis of impact of % drug loading on different formulation

**Fig. 5:**
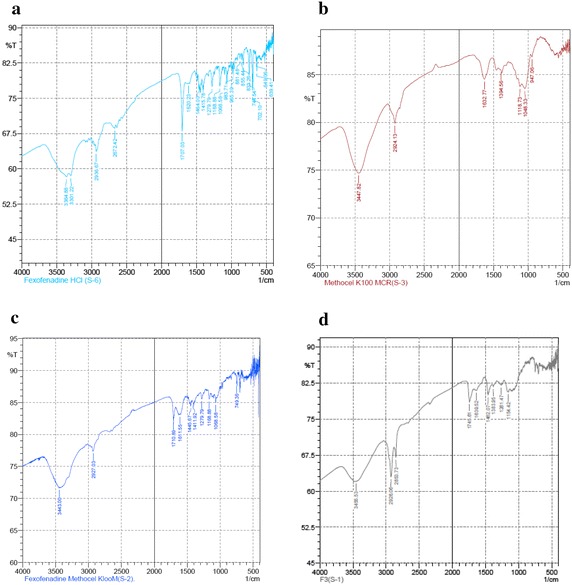
FTIR spectra of **a** pure Fexofenadine HCl (S6), **b** HPMC K100 M CR (S3), **c** physical mixture of Fexofenadine HCl and HPMC K100 M CR (S2) and **d** Fexofenadine HCl microsphere prepared with HPMC K100 M CR (F3)

Table 5 reveals that, decrease in drug loading has no significant effect on % yield but has significant increasing effect on % drug entrapment efficiency. The time required for 50 % of drug release (T_50%_) increases with increase in the total amount of HPMC K100 M CR (decrease in drug loading). Statistically it shows that, with increase in the total amount of HPMC K100 M CR by 1 mg, T_50%_ increases by 0.0032752 h. Considering the level of significance 0.05, P value has been found to be 0.000314 for formulations F1–F5. This indicates that, P value is <0.05. So it can be concluded that, the effect of the change in the amount of HPMC K 100 M CR on T_50%_ is statistically significant (Fig. 5).

**Table 5:** Summary of impact analysis of % drug loading on different formulation parameters of formulations F1–F5 using simple linear regression model (level of significance 0.05)

Formulation parameters	Regression coefficient (β)	P value	Result	Comment
Yield (%)	0.04153	0.10648	P value >0.05	Statistically insignificant
Drug entrapment efficiency (%)	0.037828	0.000848	P value <0.05	Statistically significant
Time required for 50 % of drug release (T_50%_)	0.0032752	0.000314	P value <0.05	Statistically significant

### Fourier transform infrared spectroscopy (FTIR)

**Fig. 6:**
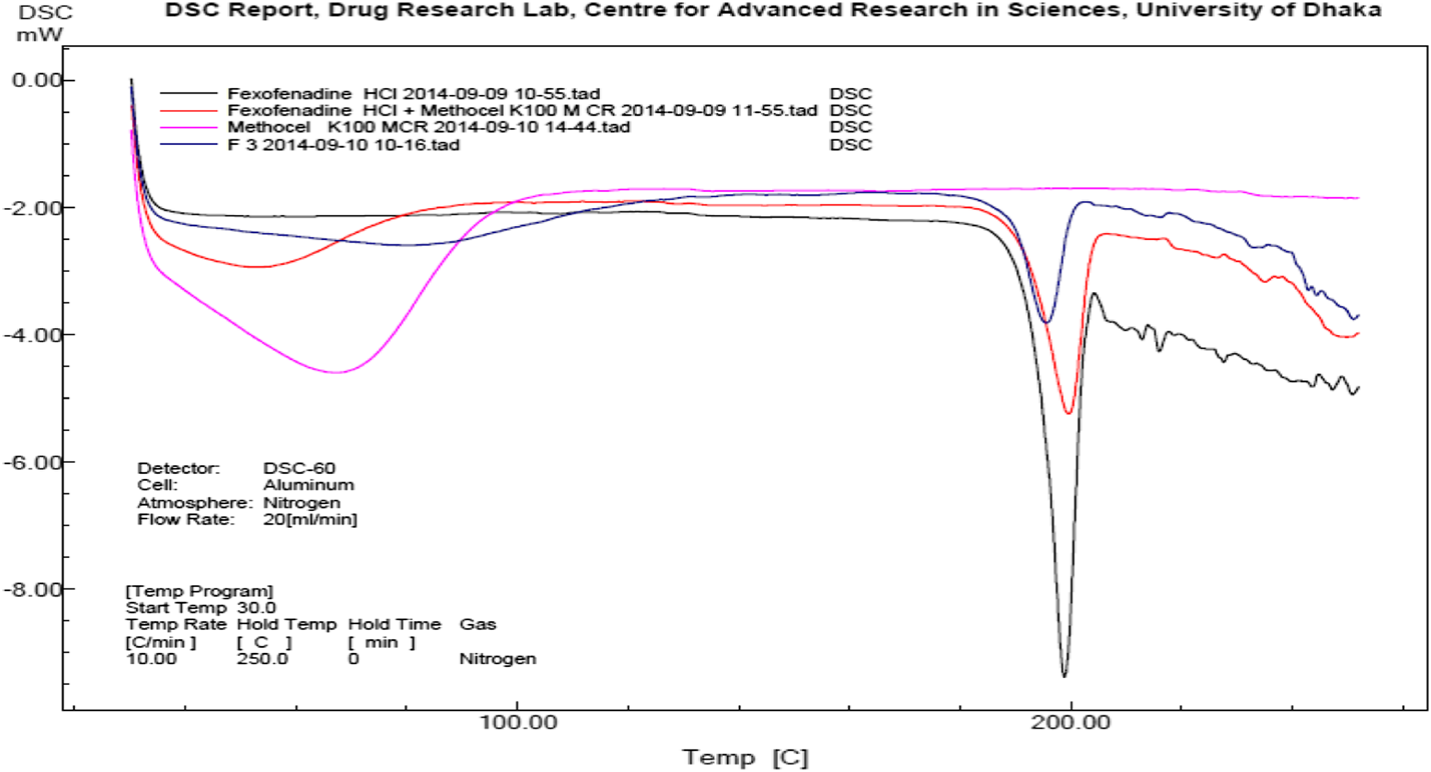
Combined DSC thermogram of pure Fexofenadine HCl, HPMC K100 M CR and their physical mixtures and F3 (microspheres of drug prepared with HPMC K100 M CR)

FTIR spectra of pure Fexofenadine HCl (S6), physical mixture of Fexofenadine HCl and HPMC K100 M CR (S2) and Fexofenadine HCl microspheres prepared with HPMC K100 M CR (F3) were observed and shown in Fig. 5. The FTIR spectra of pure Fexofenadine HCl depict characteristic absorption band at 3364.88, 1707.03, 1464, 1279.79 and 1168.88 cm^−1^ which represent the presence of broad, O–H stretching vibrations, carbonyl (C=O) stretching of Carboxylic acid, aromatic C=C stretching and C–O stretching vibration of tertiary alcohol respectively. According to Fig. 5, the FTIR spectra of Fexofenadine HCl loaded microsphere (F3) show characteristic absorption band at 3364.88, 1707.03, 1464, 1279.79, and 1168.88 cm^−1^ which represent the presence of broad, O–H stretching vibrations, carbonyl (C=O)stretching of carboxylic acid, aromatic C=C stretching and C–O stretching vibration of tertiary alcohol respectively (Table 4). It indicates no change of functional groups (Fig. 6).

### Differential scanning calorimetric (DSC) study

DSC studies were conducted for pure Fexofenadine HCl, HPMC K100 M CR and their physical mixtures and F3 (microspheres of drug prepared with HPMC K100 M CR). The data obtained from all of these samples are viewed in Fig. 6 as combined thermogram of drug, polymer and microspheres prepared with HPMC K100 M CR. No drastic change occurred to the melting point of the microspheres in comparison with pure Fexofenadine HCl. So it can be said there is no such incompatibilities in the physical mixture or in the microspheres prepared with HPMC K100 M CR in comparison to pure Fexofenadine HCl.

## Conclusion

Polymeric microspheres of Fexofenadine HCl were prepared successfully by emulsification-solvent evaporation technique using K100 M CR. In this study, it has been demonstrated that HPMC K100 M CR microspheres containing Fexofenadine HCl can be an excellent candidates for consideration in drug delivery system with a sustained effect. As the results suggest, F5 can be considered as the best formulation.

## References

[CR1] Ayon NJ, Hasan I, Islam MS, Reza MS (2014). Preparation and characterization of gliclazide incorporated cellulosic microspheres: studies on drug release. Compatibility and micromeritics. Dhaka Univ J Pharm Sci.

[CR2] Bolourtchian N, Karimi K, Aboofazeli R (2005). Preparation and characterization of ibuprofen microspheres. J Microencapsul.

[CR3] El-Kamel A, Al-Shora DH, El-Sayed YM (2006). Formulation and pharmacodynamic evaluation of captopril sustained release microcapsules. J Microencapsul.

[CR900] Higuchi T (1963). Mechanism of sustained-action medication. Theoretical analysis of rate of release of solid drugs dispersed in solid matrices. J Pharm Sci.

[CR5] Jalil R, Nixon JR (1989). Microencapsulation using poly (l-lactic acid) I: microcapsule properties affected by the preparative technique. J Microencapsul.

[CR6] Lumay G, Boschini F, Traina K, Bontemp S, Remy JC, Cloots R, Vandewalle N (2012). Measuring the flowing n-properties of powders and grains. Powder Technol.

[CR7] Manekar NC, Puranik PK, Joshi SB (1992). Microencapsulation of propranolol hydrochloride by the solvent evaporation technique. J Microencapsul.

[CR8] Panchaxari MD, Amit MB, Vinayak SM, Anand PG, Vivek WS, Prashant SS (2013). An improvement of the efficacy of moxifloxacin HCl for the treatment of bacterial keratitis by the formulation of ocular mucoadhesive microspheres. Sci Pharm.

[CR9] Parida KR, Panda SK, Ravanan P, Roy H, Manickam M, Talwar P (2008). Microparticles based drug delivery systems: preparation and application in cancer therapeutics. Int Arch Appl Sci Technol.

[CR10] Patel JK, Patel MM (2007). Stomach specific anti-helicobacter pylori therapy: preparation and evaluation of amoxicillin-loaded chitosan mucoadhesive microspheres. Curr Drug Deliv.

[CR11] Product Information: Allegra, Fexofenadine. Hoechst Marion Roussel, Kansas City (PI revised 6/98) reviewed 1/2000

[CR12] Product Information: Allegra^®^, Fexofenadine. Aventis Pharmaceuticals, Kansas City (PI revised 11/2000) reviewed 10/2001

[CR13] Raut NS, Somvanshi S, Jumde AB, Khandelwal HM, Umekar MJ, Kotagale NR (2013). Ethyl cellulose and hydroxypropyl methyl cellulose buoyant microspheres of metoprolol succinate: influence of pH modifiers. Int J Pharm Investig.

[CR14] Raval JP, Naik DR, Patel PS (2011). Preparation and evaluation of cellulose acetate butyrate microspheres containing diclofenac sodium. Int J Drug Formul Res.

[CR15] Uddin MR, Rashid MM, Alam MR (2013). In vitro evaluation of oral extended release drug delivery system for trimetazidine dihydrochloride using methocel polymers. Int J Pharm Pharm Sci.

